# Cut‐points and gray zones: The challenges of integrating Alzheimer's disease plasma biomarkers into clinical practice

**DOI:** 10.1002/alz.70113

**Published:** 2025-03-27

**Authors:** Jemma Hazan, Kathy Y. Liu, Jeremy D. Isaacs, Robert Howard

**Affiliations:** ^1^ Division of Psychiatry University College London London UK; ^2^ St George's University Hospitals NHS Foundation Trust London UK; ^3^ Neuroscience & Cell Biology Research Institute, St George's School of Health and Medical Sciences City St George's, University of London London UK

**Keywords:** Alzheimer's disease, amyloid, biomarker, blood, dementia, plasma, test

## Abstract

Plasma biomarkers for Alzheimer's disease (AD), such as plasma phosphorylated (p)‐tau217, offer a more accessible means of testing for the presence of AD pathology compared to cerebrospinal fluid (CSF) or positron emission tomography (PET) methods. They can support diagnostic assessment and determine patient eligibility for treatment with amyloid beta–lowering drugs in community settings where access to CSF examination and amyloid‐PET are limited. However, there are important challenges associated with interpreting and integrating plasma biomarker results in clinical practice. This article explores different approaches to interpreting plasma biomarker results in secondary care, important potential sources of uncertainty, and considerations for their clinical application.

**Highlights:**

- Plasma biomarkers such as phosphorylated tau‐217 (p‐tau217) offer a promising, accessible alternative to cerebrospinal fluid (CSF) and positron emission tomography (PET) for detecting Alzheimer's disease pathology, especially in settings with limited diagnostic resources.
- Clinical integration of plasma biomarker testing presents challenges, particularly in interpreting results. This includes uncertainties around intermediate results and their role in patient management.
- Clear frameworks and guidelines are essential to optimize the use of plasma biomarkers, supported by further research and education to ensure effective application in clinical practice.

## 1 INTRODUCTION

Access to cerebrospinal fluid (CSF) and amyloid positron emission tomography (PET) investigations for Alzheimer's disease (AD) is limited in many parts of the world, particularly in low‐ and middle‐income countries, including some countries in Africa, South Asia, and Latin America.^1^ These regions have variable access to the infrastructure, trained professionals, and financial resources necessary to implement these diagnostic methods.^2^

Even in some high‐income countries, such as the United Kingdom (UK) and Spain, CSF and PET use is low, particularly in community health care settings.^3^ As a case example, in the UK, most diagnoses of AD dementia are made by memory services provided in community settings by National Health Service (NHS) mental health services. In the current NICE (National Institute for Health and Care Excellence) dementia clinical guideline,^4^ specialist investigations such as PET and CSF biomarkers, are either not routinely available (PET) or not required (CSF) for establishing a dementia diagnosis and are therefore requested in only a very small minority of patients.^5^, ^6^, ^7^ As a result of deploying pragmatic, clinically‐based criteria for dementia diagnosis, the UK has not required large‐scale CSF and amyloid‐PET testing capacity. Most memory service clinicians report low levels of personal confidence when ordering or interpreting these tests,^7^ highlighting the need for a considered approach to ensure their effective use if access to biomarker information is expanded.

Recently, putative disease‐modifying therapies (DMTs) for AD that target cerebral amyloid beta (Aβ) have received regulatory approval in the United States, Japan, China and South Korea, and the UK, with a final NHS funding decision pending.^8^, ^9^ Use of these drugs requires biomarker‐supported confirmation of amyloid pathology. This presents a challenge to health care systems such as the NHS, that have not previously required large‐scale access to lumbar puncture and PET for patients in the dementia diagnosis and treatment pathway.

AD plasma biomarkers offer a less invasive, cost‐effective, and scalable means of testing for the presence of AD pathology in community memory services.^10^ Plasma biomarkers show varying sensitivity and specificity in identifying the presence of amyloid pathology, with phosphorylated tau‐217 (p‐tau217) consistently demonstrating superior specificity and sensitivity compared to other markers like Aβ42/40 or p‐tau181.^11^ Plasma phosphorylated tau (p‐tau) is a sensitive biomarker for amyloid and tau neuropathology.^12^ Plasma p‐tau species, such as p‐tau217, have shown performance comparable to that of CSF in research cohorts in distinguishing between healthy controls, preclinical AD, other neurodegenerative disorders and AD with cognitive impairment.^13^, ^14^, ^15^ Plasma p‐tau has also shown strong agreement with established amyloid‐PET and CSF fluid biomarkers.^16^, ^17^ However, data from even the most highly performing plasma markers show a greater degree of overlap in test results between amyloid‐PET‐positive and ‐negative individuals than is seen with CSF.^16^ These plasma tests serve primarily as markers of amyloid and tau neuropathology, identifying biologically‐defined AD, which is distinct from the clinical syndrome of Alzheimer's dementia.^18^ In memory services, the projected use of biomarker results may include several scenarios: (1) providing additional information as part of the diagnostic assessment, (2) selecting individuals for DMTs, and (3) evaluating response to treatment. The indication for ordering the test may vary based on its intended use and will influence its interpretation.

The integration of plasma biomarkers into routine clinical care will consequently present several challenges for clinicians, patients, and wider health care systems. Some of these challenges will include determining diagnostic cut‐points for plasma biomarker results, and communicating and managing diagnostic uncertainty and how best to use resources when a result is neither clearly positive or negative, that is, falls in an intermediate or “gray” zone.

This article explores how clinicians in secondary care, with limited experience in using AD biomarker investigations, can best understand plasma biomarker results, particularly when an intermediate result is received. It also addresses navigating the complexities involved in translating this new technology into clinical practice.

### 1.1 Establishing cut‐points for plasma biomarker results

AD plasma biomarker assays detect and provide a quantitative measure of AD pathology that lies along a continuous scale. An optimal cutoff value or “cut‐point” to dichotomize the test result as positive or negative for biological AD is determined externally, introducing potential variation in how a binary positive or negative result is defined. A summary of the key terms that are important in understanding diagnostic test performance are provided in Table 1.^19^, ^20^, ^21^

**TABLE 1:** Summary of terms.

Term	Description
Sensitivity (True positives)	The proportion of patients with a positive test result in a group of patients with the disease, that is, “true positives.”^19^
Specificity (True negatives)	The proportion of patients with a negative test result in a group of patients without the disease, that is, “true negatives.”^19^
False positives (1 – specificity)	The proportion of people who do not have a disease but are incorrectly tested positive.^20^
False negatives	The proportion of people who have the disease but are incorrectly tested negative.^20^
Positive predictive value	The ratio of true positives to the total number of positive test results, and the likelihood that a person who tests positive has the disease^21^
Negative predictive value	The ratio of true negatives to the total number of negative test results, and the likelihood that a person who tests negative does not have the disease.^21^
Pre‐test probability	The probability of the person having the disease before the test.
Post‐test probability	The probability of the patient having the disease after the test.

Sensitivity and specificity are intrinsic diagnostic accuracy properties of a test, normally obtained from a specific cohort (or cohorts) of patients selected to represent the target population. In contrast, positive predictive value (PPV) and negative predictive value (NPV) are post‐test probabilities derived from sensitivity and specificity values that reflect the likelihood of a disease given a positive or negative test result. PPV, NPV, and more recently proposed sensitivity and specificity, are all influenced by the pre‐test probability of the disease in the population being tested.^22^, ^23^

As tests in medicine are rarely 100% sensitive or specific, choosing a cut‐point will always involve a trade‐off between the sensitivity and specificity associated with the chosen value, and the consequent balance between false negatives and false positives. Each cut‐point will also have a level of uncertainty associated with its application, the size of which can be defined using confidence intervals (CIs).^24^

Currently there are no globally accepted cut‐points for plasma biomarkers such as p‐tau. Different cut‐points have been proposed, each based on varying units of measurement and studied in different populations.^11^, ^25^, ^26^, ^27^ This highlights the urgent need for a standardized approach, similar to the Centiloid scale used in amyloid‐PET, to promote consistency across populations and support clinical decision‐making. The lack of standardization poses significant challenges for diagnostic assessments in secondary care, as results may differ widely depending on the chosen threshold. The Alzheimer's Association is expected to publish recommendations on blood biomarker use in 2025, which may provide more guidance on standardized approaches.^28^

#### 1.1.1 How have cut‐points been generated for a plasma biomarker test, such as p‐tau217?

A research cohort is typically characterized by amyloid‐PET, whereby participants are categorized into binary amyloid positive (Aβ+) and amyloid negative (Aβ–) groups based on a single defined cut‐point from PET images. Plasma p‐tau217 samples for each participant are analyzed in a regression model, using plasma p‐tau217 as a predictor candidate for Aβ‐PET positivity (the reference standard). This risk–prediction regression model may include other predictor factors (e.g., apolipoprotein E (*APOE*) genotype or age).^25^ Receiver‐operating characteristic (ROC) curve analysis is then used to determine the predictive accuracy of plasma p‐tau217 in determining a binary outcome, such as Aβ‐PET positivity, by mapping the sensitivity (true positive rate), versus (1 – specificity) (false‐positive rate) for all possible values of the cut‐point between Aβ+ and Aβ– groups. The area under the curve (AUC) can then be calculated (values ranging from 0 to 1, with 1 representing perfect discrimination ability between positive and negative cases) to assess the overall predictive performance of a continuous marker.^29^

### 1.2 Single cut‐point approach

**FIGURE 1:**
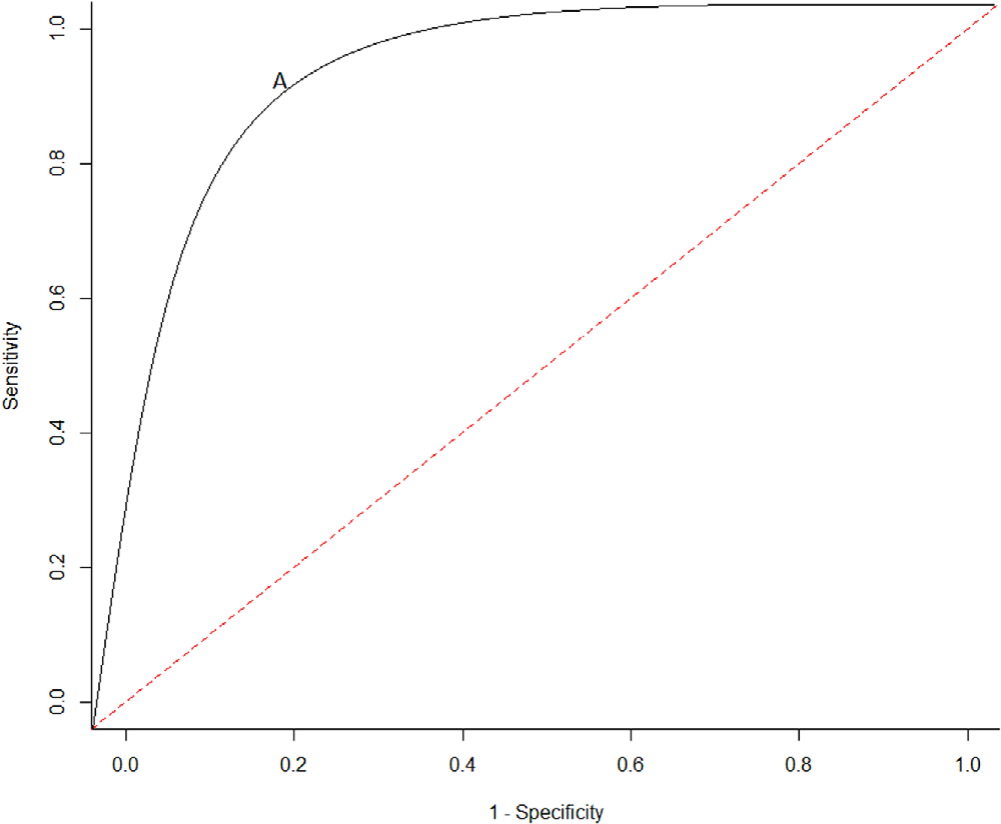
A hypothetical ROC (receiver‐operating characteristic) curve. This links data points by plotting 1 – specificity (representing false‐positive rate) on the x‐axis and sensitivity on the y‐axis, capturing all cutoff values derived from test results. The Youden index is represented by point A. The 45° red dashed diagonal line represents the ROC curve of random classification.

There are several possible approaches to generating a single cut‐point for a continuous plasma biomarker result.^24^ One approach is to calculate the Youden index, which defines the optimal cut‐point for diagnostic accuracy as the maximum point of the Youden function, representing the difference between the true positive rate and the false positive rate across all potential cut‐point values (Figure 1). The Youden index involves finding the cut‐point that maximizes the summation of sensitivity and specificity – 1,^30^ and corresponds to a point on the ROC curve with the highest vertical distance from the 45° diagonal line (Figure 1). CIs can be calculated for the Youden index, as well as for the sensitivity and specificity values used to compute it. This single cut‐point approach benefits from being more straightforward to interpret and has a clear binary outcome. However, difficulties arise with this approach where there is overlap between test results in those with and without the disease or overlap in 95% CIs for test positivity and negativity. Results located within these areas of overlap will be interpreted as indeterminate and may require further confirmatory testing.

### 1.3 Two cut‐point approach

It has been proposed recently that a two cut‐point diagnostic workflow system for plasma p‐tau217 levels may be a more efficient way of operationalizing a plasma biomarker, alongside ongoing but reduced use of CSF or PET.^25^ This method aims to maximize the PPV and NPV of the test, reduce the number of false positives and false negatives, and identify a sub‐group who require confirmatory testing. Consequently, this would be more cost‐effective than using CSF or PET in all patients.^31^

**FIGURE 2:**
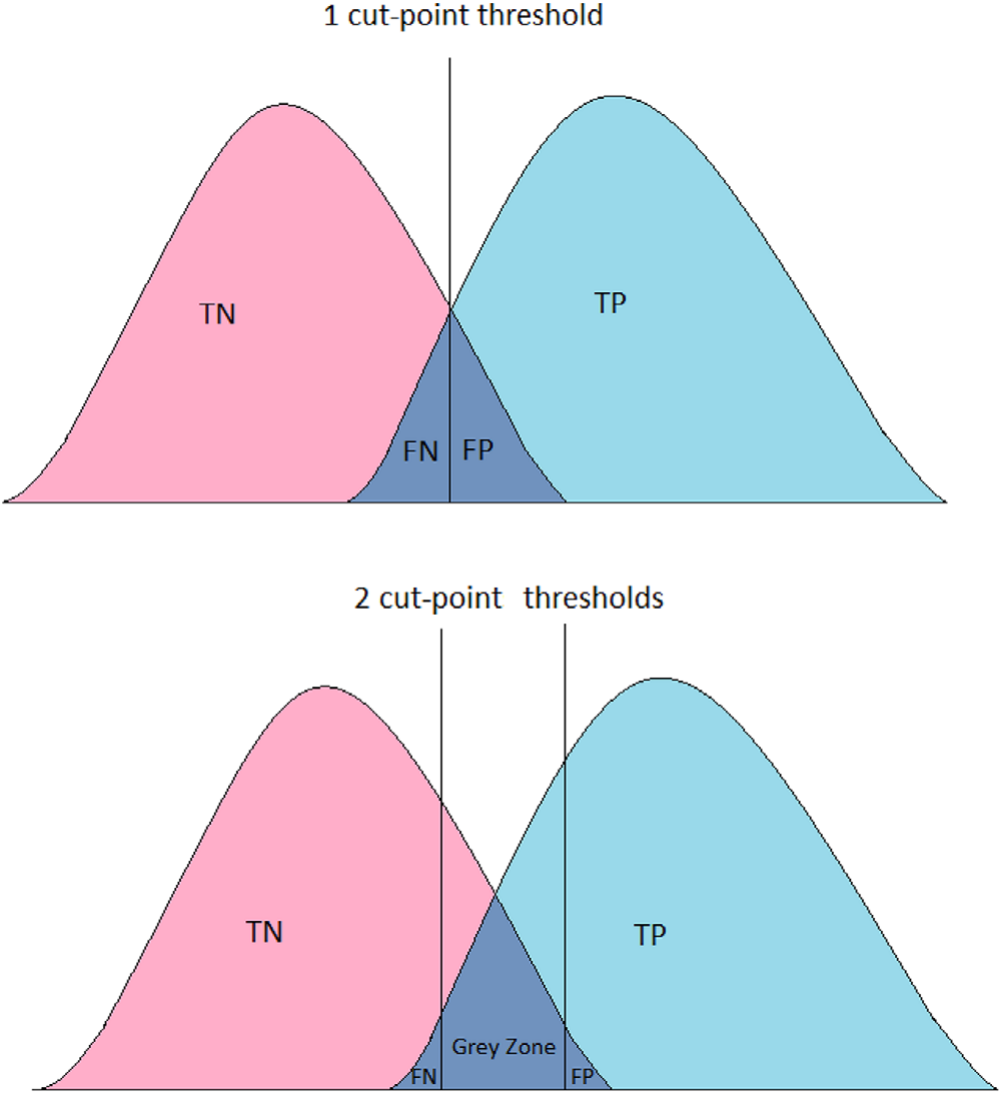
Graphical illustration of two hypothetical distributions for patients with or without amyloid bet (Aβ) pathology. (A) The vertical line indicates the cut‐point threshold to determine the presence of Aβ pathology. (B) The vertical lines indicate the two cut‐point thresholds to determine the presence of Aβ pathology, with the intermediate risk “gray zone.” TN, true negative; TP, true positive; FN, false negative; FP, false positive.

This two cut‐point approach involves the definition of lower (e.g., 90%, 95%, or 97.5% sensitivity) and higher (e.g., 90%, 95%, or 97.5% specificity) probability thresholds for Aβ PET positivity.^10^, ^11^, ^25^ Brum et al. analyzed a logistic regression model using plasma p‐tau217, age, and *APOE* ε4 as predictor candidates for Aβ‐PET positivity.^25^ Ashton et al. used a linear regression model with plasma p‐tau217, adjusting for age and sex as a predictor for Aβ‐PET positivity.^11^ They then categorized the results into three groups, with low, intermediate, or high risk of Aβ PET positivity. This enables a lower sensitivity threshold and a higher specificity threshold than would be provided by a single cut‐point. A higher sensitivity threshold will reduce the number of false‐negative results (i.e., avoid incorrectly labeling patients with AD pathology as Aβ negative). Similarly, a higher specificity threshold would reduce the number of false‐positive results (i.e., avoid classifying patients who do not have AD pathology as Aβ positive). The one cut‐point versus two cut‐point threshold approach is presented pictorially in Figure 2.

The two cut‐point approach introduces an intermediate risk or “gray” zone, with higher sensitivity or specificity thresholds increasing the magnitude of this zone. Brum et al. reported that the proportion of participants in this intermediate risk zone ranged from 13.5% to 41.0%, depending on how stringently the thresholds were set.^25^ Ashton et al. employed 95% sensitivity and specificity thresholds and found that the intermediate risk zone ranged from 13.0% to 22.9%, which they proposed related to the varying degree of Aβ‐positivity prevalence in the different cohorts.

### 1.4 Confirmatory testing

A proposed solution for patients with an intermediate risk result on plasma biomarker testing is referral for confirmatory testing with “gold standard” investigations, such as CSF or amyloid‐PET. Single cut‐points are used in AD CSF marker testing and amyloid‐PET.^32^ It is important to note that even these investigations do not have 100% sensitivity and specificity, and can sometimes show discordance with each other and post‐mortem neuropathology.^33^ The threshold for amyloid‐PET positivity, often considered the “standard of truth” for the presence of AD pathology, varies across studies and can range from 13.5 to 24 Centiloids.^11^, ^34^, ^35^ This variability in the amyloid‐PET positivity threshold directly impacts the reported accuracy of plasma biomarkers, as these thresholds are typically used as reference points for evaluating biomarker performance. Regulatory approval of CSF assays was based on agreement with positive/negative visual interpretation of amyloid PET scans. The sensitivity/specificity of approved CSF assays against this standard ranged from 97%/84% to 88%/92%.^36^, ^37^

There are limitations to using a single cut‐point in amyloid‐PET imaging, such as its susceptibility to variations in acquisition methods and the specific cut‐point applied.^38^, ^39^ Discrepancy between a positive fluid biomarker (CSF/blood) and a negative PET result can arise where fluid biomarkers become positive earlier in the disease course than PET imaging changes.^40^ However, fluid p‐tau levels have been shown to change around the same time as amyloid‐PET.^41^ Further studies are needed to test the concordance between intermediate plasma results and subsequent CSF or amyloid‐PET testing in representative community cohorts.

Because as currently conceived, a two cut‐point approach requires a significant minority of patients to undergo confirmatory CSF or amyloid‐PET testing, provision of these investigations will still have to be expanded. This will involve associated assay or radiotracer costs, procurement of space and equipment, and staff training. In the likely event that any one of these is in limited supply, a decision to refer a patient for confirmatory testing will result in a prolonged wait for patients to receive a diagnosis or treatment.^42^

### 1.5 Alternative approaches to confirmatory testing

If blood biomarker testing is considered the only scalable option, clinicians and regulators must determine the level of plasma assay accuracy required to eliminate the need for CSF or PET in the evaluation of memory clinic patients. One proposal has been that the blood test should achieve a performance comparable to CSF tests, with a sensitivity and specificity of ≈90%.^43^ Superior test accuracy will be crucial for selecting individuals for DMTs, where the importance of establishing an unequivocal biological diagnosis of AD is most obvious.

If plasma assays have a diagnostic performance equivalent to approved CSF assays, one approach to an intermediate risk result could be to decide that no further confirmatory testing is employed. In such a scenario, the clinician would accept the same level of diagnostic uncertainty that currently exists with CSF testing. Formal neuropsychological testing may serve as an alternative to support clinical interpretation in cases with intermediate or inconclusive biomarker results. However, it is unlikely to significantly reduce diagnostic waiting times, as access to these services remains a limited resource in many health care settings.^44^

Another option, using the two cut‐point approach, would be that only those who unambiguously fall in the high‐risk group would be eligible for Aβ lowering treatment, whereas those with negative or intermediate risk results will not. It is widely recognized that only a small fraction of patients will be eligible to receive these treatments.^45^ This process may therefore function primarily as a means of determining eligibility rather than initiating further extensive testing.

Those patients with an intermediate risk result could be offered more intensive surveillance, with potential repeat plasma biomarker testing, although data are currently lacking to suggest an appropriate testing interval. Prior work has shown that in cognitively unimpaired participants who undergo longitudinal plasma biomarker testing, there are associations between change in plasma biomarker concentrations and future risk of neurodegeneration and cognitive decline.^46^

### 1.6 Pre‐counselling

Clinicians will need to convey the diagnostic and therapeutic implications of receiving an intermediate risk result to patients in a clear and comprehensive way through shared decision‐making.^42^ This will ensure patients are adequately pre‐counseled on the risk of such a result and can provide informed consent for testing. If deemed clinically appropriate, the clinician will then need to assess if the patient is willing to participate in further confirmatory testing. Amyloid‐PET requires the patient to lie still for an extended period and is associated with radiation exposure.^47^ Lumbar puncture (LP) for CSF testing is invasive, contraindicated in certain bleeding disorders, requires cessation of anticoagulant and some anti‐platelet medications, and can be associated with post‐LP headache.^48^ Alternatively, if the patient and clinician are willing to accept the possibility of an intermediate result with no further confirmatory testing, investigation will stop at this point. Consideration of clinical factors, including the pre‐test probability, will influence if it is appropriate to order the plasma biomarker test in the first place.

### 1.7 Pre‐test probability

It is important to distinguish between the sources of pre‐test probability and their impact on the interpretation of test results. Pre‐test probability, or the likelihood that a person has the disease before the test, is influenced by the prevalence of the disease and the outcome of a clinical assessment that considers symptoms, age, genetic risk, and so on. Prevalence refers to the overall rate of a disease in a population, with higher prevalence increasing the pre‐test probability that an individual has the disease, which is particularly relevant in disease‐screening situations.

In contrast, when a test is used for diagnostic confirmation or to decide treatment eligibility, a clinical assessment will evaluate a patient's symptoms and history and estimate the likelihood of disease. In this context, prevalence sets the baseline likelihood of disease, while clinical assessment independently refines this probability based on individual patient factors. Even in memory clinic populations with a high overall prevalence of AD, the individual clinical assessment may result in a low pre‐test probability.

Thus, the pre‐test probability is likely to differ depending on the point in the clinical pathway that the blood test is deployed, that is, before or after clinical assessment in a secondary service. If used at an earlier stage in the pathway, (e.g., in the general population prior referral to a memory clinic), the prevalence and pre‐test probability of biological AD might be lower than 50%.^49^ Blood biomarkers for AD typically demonstrate lower accuracy in detecting pathology among cognitively normal individuals from general population samples compared to clinical cohorts, primarily due to the lower prevalence of disease in these populations.^26^ The prevalence of AD is likely to be high in memory services, which serve an older population (often over 80 years of age) who present with cognitive (generally memory) complaints. If after clinical assessment a clinical diagnosis of AD is considered likely, the pre‐test probability of AD pathology will be higher, potentially around 80%.^50^

Even with high (but less than 100% perfect) diagnostic accuracy, the negative and positive predictive performance of the test depends on the pre‐test probability in the group of patients tested, which in turn can be influenced by the prevalence or clinical assessment outcome.^51^ Plasma p‐tau217 has been reported to have a lower PPV than NPV, even in memory clinic samples where amyloid positivity is more prevalent^11^ and especially in samples with a lower prevalence of amyloid positivity.^26^ A higher pre‐test probability increases the probability that a positive result is a true positive (the PPV) but lowers the probability that a negative result is a true negative (NPV). If the pre‐test probability is 80%, a blood biomarker test with 90% accuracy has a PPV of 97% and a NPV of 69%.^43^ Thus, in cases where there is already higher clinical confidence of an AD diagnosis, a positive test would help confirm the clinical diagnosis (and eligibility for DMTs) as it is less likely to be a false positive, but a negative test cannot confidently rule it out as it is more likely to be a false negative.^43^ Conversely, a lower pre‐test probability increases the NPV but decreases the PPV. If there is low clinical expectation of an AD diagnosis (pre‐test probability 20%), a negative test result with 90% accuracy could help to confirm a non‐AD diagnosis (NPV 97%) but a positive test result does not confidently attribute the cause of the symptoms to AD (PPV 69%). In this scenario, clinicians should apply caution in making an AD diagnosis (and ascertaining eligibility for DMTs) based on a single positive test result if this is not supported by the clinical assessment. Without a high post‐test probability of having AD, there is the potential for iatrogenic harm from labeling people as having AD and subsequent exposure to DMTs when there could be an alternative cause for their symptoms. Where there is a mismatch between the clinical assessment and the test result, it is sensible to perform a second test to lower the chance of the first result being a false negative or a false positive.

**FIGURE 3:**
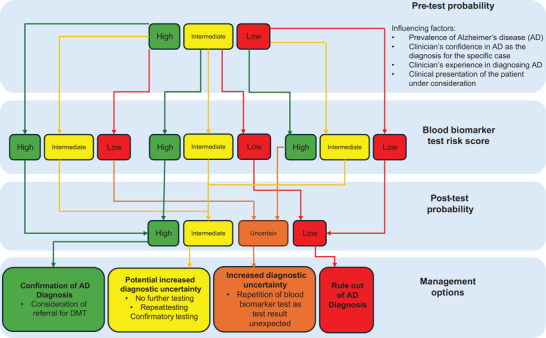
Flowchart of factors influencing pre‐test probability, post‐test probability, and subsequent management options.

In memory clinics, blood biomarkers are most useful as diagnostic confirmatory tests when the clinician is uncertain about the AD diagnosis, that is, an intermediate pre‐test probability of around 50%. In these cases, a positive result from a test with 90% accuracy will have a greater impact on making an AD diagnosis and determining eligibility for DMTs (PPV 90%), and a negative test result will help to rule out AD as a contributing pathology (NPV 90%). The possible process and implications of using a blood biomarker test for diagnosing AD are illustrated in Figure 3.

### 1.8 Additional considerations for test result interpretation

Clinicians may face other challenges when interpreting plasma p‐tau in clinical practice. A key issue is the high prevalence of amyloid positivity in cognitively normal elderly individuals, which increases with age—reaching 41.3% in those 80–89 years of age.^52^ In such cases, amyloid may act as a potential predictor of cognitive impairment that may never develop during the patient's lifetime, rather than a pathological diagnosis of dementia, reducing the PPV of p‐tau. Clinicians will need to understand that amyloid deposition occurs in cognitively unimpaired individuals, who often will not live long enough to develop dementia.

Furthermore, up to 50% of dementia cases with Alzheimer's pathology involve mixed etiologies and are often accompanied by vascular co‐pathologies or conditions such as Lewy body disease.^53^, ^54^ Clinicians should be equipped with the knowledge to interpret biomarker results within the context of these complex, overlapping pathologies.

Interpreting AD plasma biomarker results will require careful consideration of demographic factors and the presence of other comorbidities, which are more prevalent in memory service patients.^26^ Renal function may influence plasma p‐tau levels, although studies have reported mixed results regarding the association between chronic kidney disease (CKD), lower estimated glomerular filtration rates (eGFRs), and higher p‐tau181, p‐tau217, and unphosphorylated tau concentrations^55^, ^56^ Similarly, the relationship between body mass index (BMI) and p‐tau levels remains unclear, with some findings suggesting an inverse association, possibly due to a dilutional effect.^57^ Sex differences in blood biomarker levels have been observed, but findings vary across studies.^58^, ^59^

These factors highlight the importance of tailoring biomarker interpretation to individual clinical profiles. Clinicians will need training on how these factors influence p‐tau levels, particularly when faced with unexpected results. Future developments may include online risk score calculators using large cohort data to provide decision‐support in result interpretation.^60^, ^61^

The potential shift from multidisciplinary to single‐clinician decision‐making that is likely to accompany the adoption of AD blood biomarkers may introduce several challenges. Individual clinicians will bear greater responsibility for interpreting results without team input, which could lead to variability, particularly when the results conflict with the clinical impression. This issue may be more pronounced with referrals from primary care or private settings, where secondary care clinicians might receive p‐tau results as part of the referral. In such cases, clinicians will have to interpret and communicate test results they did not order, and that they may not have deemed appropriate after their specialist assessment, with the potential to complicate the diagnostic process. Establishing clear protocols on who should order these tests will be crucial.

Communicating conflicting findings (where the history and examination point one way but the biomarker suggests something different) to patients will also require careful consideration, and will necessitate nuanced discussions. To address these challenges, solutions include comprehensive clinician training, standardized diagnostic guidelines, decision‐support tools for use of biomarkers, continued access to multidisciplinary expertise for complex cases, and patient and caregiver education resources.

The implementation of plasma biomarkers in clinical practice may, over time, lead to situations where previous diagnoses based on predominantly positive biomarker data or clinical symptoms alone require re‐evaluation. This could also result in the need for more precise diagnostic labels, for example, recognizing that large numbers of older people will have pathological AD without symptoms that would lead to a diagnosis of AD dementia.

### 1.9 Case scenarios

The following case scenarios illustrate key topics discussed in this paper:

Case 1: Mr Johnson is a 74‐year‐old man, referred following a 6–12 month history of gradual cognitive decline, primarily affecting his episodic memory. According to the General Practioner (GP) referral, he has difficulty recalling recent events, including conversations with friends, and often misplaces items around the house. Despite these symptoms, Mr Johnson himself is not particularly concerned. The clinician is unable to obtain additional information from an informant.

During the initial evaluation, Mr Johnson scores 28/30 on the Mini‐Mental State Examination (MMSE). Based on this assessment and before conducting any further tests, the clinician estimates an intermediate probability (around 50%) of Alzheimer's disease (or AD) as the underlying cause of his cognitive symptoms. A plasma p‐tau217 test is ordered, and the result comes back as positive.

Dr Smith discusses the potential next steps with Mr Johnson, including the importance of further confirmatory testing including detailed neuropsychometry, and a magnetic resonance imaging (MRI) scan.

Outcome: The neuropsychometry confirms presence of episodic memory and visuospatial and language impairment that cannot be explained by Mr Johnson's age or educational background. The MRI scan shows bilateral hippocampal atrophy and mild cortical volume loss. The post‐test probability at this point of cognitive symptoms secondary to AD is 75%. Mr Johnson is diagnosed with dementia in Alzheimer's disease.

Case 2: Mrs Thompson is a 71‐year‐old woman referred following a 6‐month history of difficulty solving crossword puzzles and forgetting the names of people and places. Her husband, who serves as an informant, has not noticed any significant changes in her day‐to‐day functioning or memory beyond what she describes. Mrs Thompson reports that she has been taking over the counter promethazine antihistamines for the last 6 months due to hay fever symptoms.

During the initial assessment, Mrs Thompson scores 28/30 on the MMSE. Based on this assessment and before conducting any further tests, the clinician considers the diagnosis may be mild cognitive impairment and estimates an intermediate probability (around 50%) of Alzheimer's disease (or AD) as the underlying cause. A plasma p‐tau217 test is ordered, which has a result that is intermediate. An MRI scan reveals age‐related involutional changes, leaving the post‐test probability of the presence of AD pathology unchanged at around 50%.

Dr Smith explains that a diagnosis of mild cognitive impairment of uncertain etiology may be present, and that the hay fever tablets may be contributing to anticholinergic burden. They discuss the next steps in management, including acknowledgement of the current level of diagnostic uncertainty and to arrange a follow‐up cognitive assessment in 6–12 months.

Outcome: Considering Mrs Thompson's age and current clinical presentation, Dr Smith decides on a “watch‐and‐wait” approach. She schedules a follow‐up assessment and plans to repeat the cognitive examination.

Case 3: Dr Wilson evaluates Mrs Davis, an 81‐year‐old woman. She reports difficulties with remembering news items on the television and occasional problems with executive functioning, such as planning and organizing. Mrs. Davis also has a past medical history of hypertension and hyperlipidemia, which raise the possibility of a vascular contribution to her cognitive decline. She scored 22/30 on the MMSE. Based on her clinical presentation and medical history, Dr Wilson estimates a 50% pre‐test probability of Alzheimer's disease (or AD), but he also strongly suspects vascular cognitive impairment as an additional contributing factor.

To gain further certainty, Dr Wilson orders a plasma p‐tau217 test, which returns a positive result, indicating the likely presence of AD pathology. The post‐test probability at this point of her cognitive symptoms being secondary to AD is 75%. To assess the extent of a vascular contribution, an MRI scan is performed, revealing substantial vascular burden.

Outcome: Dr Wilson carefully reviews the findings with Mrs Davis, explaining that the positive plasma p‐tau217 result confirms the presence of Alzheimer's disease pathology in her brain. However, the significant vascular burden observed on the MRI suggests that her cognitive symptoms may arise from a combination of AD and vascular factors, rather than AD alone. This is consistent with a diagnosis of mixed dementia in Alzheimer's disease and cerebrovascular disease

## 2 CONCLUSION

Much of the recent AD biomarker literature has focused on the analytical and diagnostic accuracy of newly available plasma markers. Further exploration is required to understand how information from these plasma biomarkers is interpreted and integrated into both the individual patient diagnostic assessment and the wider health care system. This is currently underexplored. Although there is potential for improved diagnostic accuracy with plasma biomarkers, it is also necessary to consider cost‐effectiveness, feasibility, and capacity to tolerate potential diagnostic uncertainty. Additional scrutiny of the proposed two cut‐point approach, with its implication that ≈20% of patients who undergo a blood biomarker test might require confirmatory testing, is warranted. Confirmatory testing is likely to be inappropriate for some patients and unfeasible within some health care systems. Even if confirmatory testing is undertaken, the result could still be ambiguous if the result lies close to the cut‐point and therefore do not resolve the uncertainty. This requires further exploration using large longitudinal cohort data from representative community settings with matched plasma and CSF/amyloid‐PET. If most patients who undergo plasma testing are not appropriate for confirmatory testing, guidelines will need to be developed to assist clinicians to use plasma biomarkers as a single test.

In the meantime, as with all diagnostic tests in clinical use, clinicians and patients will need to manage a degree of associated uncertainty.^62^ The degree of uncertainty tolerated will depend on clinician factors, such as training, confidence, and experience. This will likely impact the quantity of investigations ordered.^63^ A mismatch between clinicians’ assessment and the test result represents a potential source of uncertainty when using AD biomarkers. In addition, uncertainty can stem from the plasma test results themselves when the results fall into an intermediate zone or area of overlapping CIs for test positivity and negativity. In a clinic setting, it is crucial for the clinician to evaluate if a positive or negative biomarker result is consistent with their holistic clinical assessment. In determining eligibility to receive treatment with DMTs, a high post‐test probability will be important. The development of appropriate use guidelines will support the effective adoption of this technology in settings where biomarker test use is less common, such as memory services. In addition, educational tools will be essential for training clinicians on obtaining informed consent for these tests and accurately interpreting the results.

## CONFLICT OF INTEREST STATEMENT

J.D.I. reports consultancy work for Roche and a speaker's fee from Biogen, all paid to his institution, as well as consultancy work for Nestlé Health Science and conference registration and expenses from Roche. The other authors declare no competing interests. Author disclosures are available in the Supporting Information.

## CONSENT STATEMENT

There were no human participants in this study and therefore consent was not necessary.

## Supporting information



Supporting Information
